# A Novel, Efficient Algorithm for Subsurface Radar Imaging below a Non-Planar Surface

**DOI:** 10.3390/s23229021

**Published:** 2023-11-07

**Authors:** Ingrid Ullmann, Martin Vossiek

**Affiliations:** Institute of Microwaves and Photonics, Friedrich-Alexander-Universität Erlangen-Nürnberg, 91058 Erlangen, Germany; martin.vossiek@fau.de

**Keywords:** radar, subsurface imaging, synthetic aperture radar, ground penetrating radar, MIMO radar, non-destructive testing

## Abstract

In classical radar imaging, such as in Earth remote sensing, electromagnetic waves are usually assumed to propagate in free space. However, in numerous applications, such as ground penetrating radar or non-destructive testing, this assumption no longer holds. When there is a multi-material background, the subsurface image reconstruction becomes considerably more complex. Imaging can be performed in the spatial domain or, equivalently, in the wavenumber domain (k-space). In subsurface imaging, to date, objects with a non-planar surface are commonly reconstructed in the spatial domain, by the Backprojection algorithm combined with ray tracing, which is computationally demanding. On the other hand, objects with a planar surface can be reconstructed more efficiently in k-space. However, many non-planar surfaces are partly planar. Therefore, in this paper, a novel concept is introduced that makes use of the efficient k-space-based reconstruction algorithms for partly planar scenarios, too. The proposed algorithm forms an image from superposing sub-images where as many image parts as possible are reconstructed in the wavenumber domain, and only as many as necessary are reconstructed in the spatial domain. For this, a segmentation scheme is developed to determine which parts of the image volume can be reconstructed in the wavenumber domain. The novel concept is verified by measurements, both from monostatic synthetic aperture radar data and multiple-input–multiple-output radar data. It is shown that the computational efficiency for imaging irregularly shaped geometries can be significantly augmented when applying the proposed concept.

## 1. Introduction

High-resolution radar imaging originates from Earth remote sensing (see, e.g., [1,2]). In the past decades, it was adapted to a number of close-range applications, e.g., security screening of persons [3,4] or automotive radar imaging [5,6]. In all these applications, the electromagnetic waves can usually be assumed to propagate through free space. However, there are imaging scenarios where this assumption no longer holds. These include ground penetrating radar [7], non-destructive testing [8], or through-the-wall radar [9]. In such scenarios, the waves propagate through an inhomogeneous background (e.g., air and soil in ground penetrating radar). In that case, an image reconstruction becomes more complex compared to the classical case: at the material boundary, the wave encounters a change in propagation velocity, which results in refraction for non-normal incidence. To reconstruct an image from these scenarios correctly, algorithms that take into account the above-named effects have to be applied.

An image formation can be performed in the time and space domain or in the temporal and spatial frequency domain. When using a stepped-frequency continuous wave (SFCW) radar, as is conducted in our measurements and will be assumed throughout this paper, the radar data are captured in the frequency domain. Accordingly, reconstruction algorithms can be distinguished by whether they operate in the spatial domain or in the spatial frequency domain (also termed wavenumber domain or *k*-space).

A classical reconstruction algorithm in the spatial domain is the Backprojection algorithm (refs. [3,10]). It can be applied to any kind of synthetic aperture or array but is computationally demanding. The computational efficiency decreases further for heterogeneous media. In that case, for determining the refracted wave propagation, a numerical ray tracing step has to be introduced in the reconstruction process. The ray tracer computes the refracted wave path (also termed optical path) by numerical optimization based on Fermat’s principle (see, e.g., [11]). Only for a planar material boundary, there is an analytic formulation for the refracted wave path [11].

In the late 1970s, the first reconstruction algorithms operating in the wavenumber domain were introduced in geophysics. These approaches, named Phase Shift Migration [12] and Range Migration [13], are more efficient than the Backprojection algorithm since they can reconstruct an image line by line or even altogether in one step. Accordingly, they were soon adopted for monostatic synthetic aperture radar (SAR) imaging [14] and more recently to multiple-input–multiple-output (MIMO) radar [15,16]. However, in subsurface imaging, when there is a heterogeneous background, these efficient algorithms can only be applied when there is a planar material boundary or a multi-layer scenario with several planar boundaries.

Some efforts have been undertaken to overcome this problem and use wavenumber domain migration also for geometries below an irregularly shaped surface. These include Split-Step Migration [17], Phase Shift Plus Interpolation [18,19], and others [20,21]. However, all these approaches are iterative and therefore still lack efficiency.

In short, an efficient image reconstruction of heterogeneous media can only be performed when the material boundary is planar. For reconstructing objects with a non-planar surface, up to today, often the Backprojection algorithm with ray tracing (see, e.g., [11,22]) is used, and the reconstruction of large objects is far from real-time.

**Figure 1:**
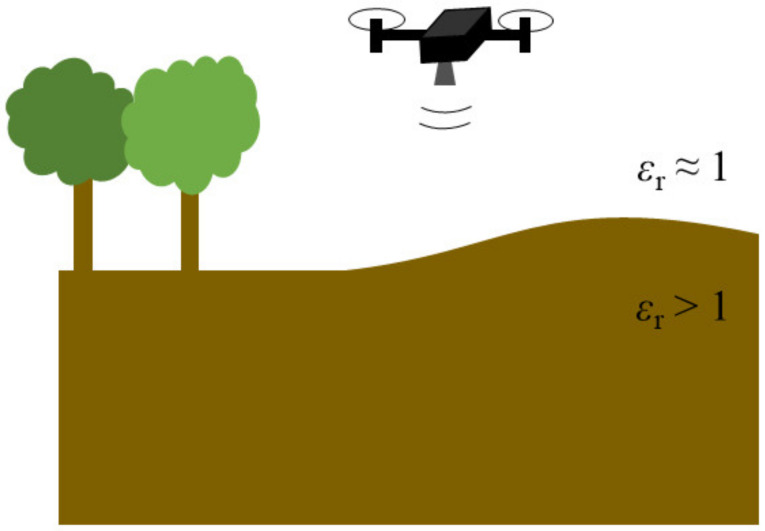
Subsurface radar imaging scenario: a drone carrying a radar to image subsurface regions below a curved planar ground surface.

However, many irregular surfaces are at least partly planar: in ground penetrating radar, this may be a ground that is flat with a few hills and troughs. Such a scenario is illustrated in Figure 1: a ground penetrating radar, carried by an unmanned aerial vehicle (UAV), investigates a terrain with an even surface adjacent to a hill. The radar is intended to help detect structures hidden below the surface. These can be man-made, such as pipelines, cables, or landmines, as well as geophysical, such as water or soil layers [23]. 

Test objects from non-destructive testing with a partly planar surface include, e.g., rotor blades.

Note that, in fact, a partly planar surface is a natural assumption when dealing with curved surfaces in microwave imaging: if the surface was completely irregular, it would be hard to reconstruct a good image even with the Backprojection algorithm for heterogeneous media. Its ray tracing step is based on geometrical optics—which, in turn, is valid only for “large” object geometries compared to the employed wavelength (typically larger than ten times the wavelength).

When the scenario under test has a surface that is partly planar, the question naturally arises whether it could be possible to use the efficient wavenumber domain reconstruction algorithms in such cases at least partly.

In this paper, we will demonstrate that, with a proper segmentation of the reconstruction domain, it is possible to reconstruct the image correctly, completing it partly in the wavenumber domain. The derivation of such an efficient and yet exact method is the goal of this work. To our best knowledge, no similar approach has been published before.

The rest of the paper is outlined as follows: in Section 2, the novel image reconstruction concept is derived. The derivation is illustrated by an example with a monostatic SAR. In Section 3, the proposed concept is extended to MIMO array imaging in the context of non-destructive material testing. Eventually, a conclusion is drawn.

## 2. Novel Reconstruction Concept

In this section, the novel concept for subsurface imaging below a non-planar surface is presented. For a better understanding, we first recapitulate imaging as a formalism. Based on this, the novel approach will be derived.

For the sake of simplicity, a monostatic radar with equidistant sampling is employed for the derivation. However, the concept can readily be extended to sparse MIMO arrays, as will be shown subsequently.

### 2.1. Imaging Formalism

Consider an electromagnetic scattering problem, linearized by the first Born approximation, for a monostatic radar system whose aperture is rectangular along the *x* and *y* directions, at *z* = 0:

(1) $$s_{\mathrm{scat}}\left(x_{A},\;y_{A},\;z\;=0,\;\omega \right)=∭o(x,y,z_{0})\exp\left\{-j2k\sqrt{({x-x_{A})}^{2}+({y-\;y_{A})}^{2}+z_{0}^{2}}\right\}\mathrm{dx}\mathrm{dy}\mathrm{dz}$$

In (1), $s_{\mathrm{scat}}\left(x_{A},\;y_{A},\;z\;=0,\;\omega \right)$ is the scattered signal at a specific antenna location, denoted by index “A”, and frequency *ω*. The object distribution is denoted $o(x,y,z_{0})$. The exponential term in (1) is the Green’s function (neglecting its amplitude), which describes wave propagation from the transceiver to the target and back to the transceiver. In it, the factor 2 accounts for the two-way propagation in the monostatic radar system and *k* denotes the wavenumber

(2) $$k=\frac{2\;\pi \;}{\lambda }=\frac{\omega }{c},$$

where *λ* is the wavelength, and *c* is the propagation velocity. When we assume free-space propagation, the wave’s propagation path in (1) equals the direct path between an antenna and a target point.

Imaging is the process of reconstructing an estimate of the object distribution from the scattered field. An estimate of the object distribution, $o^{\prime }(x,y,z_{0})$, can be obtained by solving the inverse scattering problem:

(3) $$o^{\prime }(x,\;y,\;z_{0})=\int \int \int s_{\mathrm{scat}}\left({\;x}_{A},\;y_{A},\;z\;=0,\;\omega \right)\exp\left\{+j2k\sqrt{({x-\;x_{A})}^{2}+({y-y_{A})}^{2}+z_{0}^{2}}\right\}dx_{A}dy_{A}d\omega$$

In a real-world system, the data will be captured at discrete values, which is why the formulation will read as

(4) $$o^{\prime }(x,\;y,\;z_{0})=\sum_{{\;x}_{A}}\sum_{y_{A}}\sum_{\omega }s_{\mathrm{scat}}\left({\;x}_{A},\;y_{A},\;z\;=0,\;\omega \right)\cdot \exp\left\{+j2k\sqrt{({x-x_{A})}^{2}+({y-y_{A})}^{2}+z_{0}^{2}}\right\}$$

Implementing the formulation in (4) is referred to as Backprojection. The exponential term in (4), which is the complex conjugate of the Green’s function, is the reconstruction kernel of the Backprojection algorithm. Due to the spatial variance of the reconstruction kernel, the Backprojection algorithm is very flexible but very demanding in terms of computation time. The reconstruction is performed voxel by voxel.

When there is a multi-material background, the propagation term needs to be adjusted. As the wave encounters a change in material, two effects will occur, namely a change in propagation velocity and (resulting from it) refraction. These two effects can be incorporated into the reconstruction kernel

(5) $$h\left(x,y,z\right)=\exp\left\{j2k\sqrt{({x_{B}-x_{A})}^{2}+({y_{B}-y_{A})}^{2}+z_{B}^{2}}\right\}+\;\;\;\;\;\;\;\;\;\;\;\;\;\;\;\;\exp\left\{j2k\sqrt{\varepsilon_{r}}\sqrt{({x\;-x_{B})}^{2}+({y-y_{B})}^{2}+({z-z_{B})}^{2}}\right\}$$

**Figure 2:**
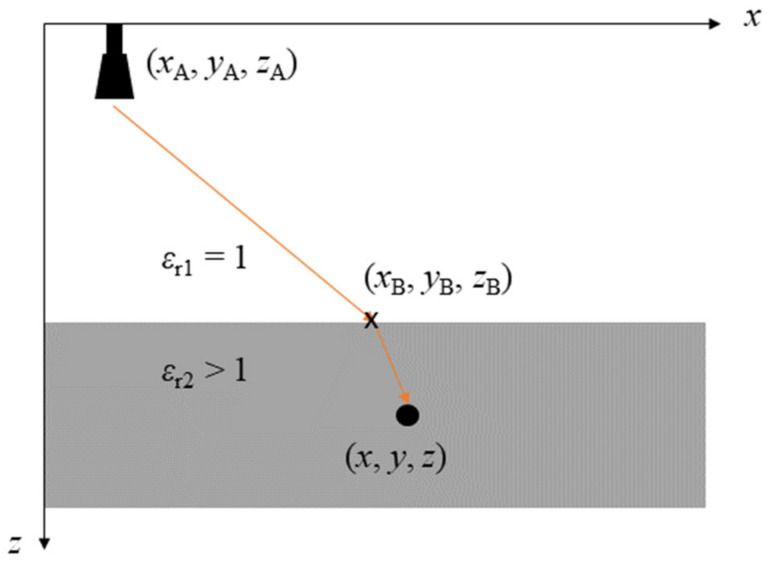
Illustration of the refracted optical path from an antenna to a target in a two-layer geometry.

The expression describes the refracted optical path as illustrated in Figure 2. Here, index “B” denotes the refraction point at the boundary. To account for the change in propagation velocity, the wavenumber *k* is scaled by the square root of the material’s relative permittivity $\varepsilon_{r}$.

To implement a reconstruction kernel as in (5), the refracted optical path, which is determined by the refraction point (*x*_B_, *y*_B_, *z*_B_), needs to be known. It can be computed by Snell’s law

*n*_1_ · sin(*α*) = *n*_2_ · sin(*β*) (6)

when the incident angle *α* is known. However, for an imaging problem, when the starting point (at the antenna) and the endpoint (the target voxel) are provided instead of the incident angle, in general, the optical path cannot be computed analytically [11]. To find it, numerical methods are required. One possibility for this is numerical ray tracing based on Fermat’s principle. For this method, first, the boundary is discretized into elements. For each element, the optical paths are computed for a respective antenna and target position. Fermat’s principle now states that a wave follows that optical path that minimizes the travel time, which is why it is also known as the “principle of least time”. Therefore, that boundary point out of the discretized values that minimizes the travel time is the true refraction point [22]. This method is called “bending”. Other possible approaches include shooting rays or so-called Eikonal solvers [24]. It is obvious that solving such an optimization problem decreases computational efficiency further still.

Equivalent to the spatial domain formulation (1), the scattering problem can be formulated in the spatial frequency domain. Again, assuming free-space propagation first, the wavenumber domain formulation of the scattering problem reads as

(7) $$S_{\mathrm{scat}}\left({k_{x}}_{\;},\;k_{y},\;\omega \right)=O\left({k_{x}}_{\;},\;k_{y},\;\;z_{0}\right)\;\text{·}\;\exp\;\left\{-j\;k_{z}\;z_{0}\right\}$$

Here, $S_{\mathrm{scat}}\left({k_{x}}_{\;},\;k_{y},\;\omega \right)$ is the receive spectrum, which can be obtained from a two-dimensional Fourier transform of the receive data $s_{\mathrm{scat}}\left(x_{A},\;y_{A},\;z\;=0,\;\omega \right)$ w.r.t. *x* and *y*. The terms *k_x_* and *k_y_* denote the spatial wavenumbers. Again, neglecting amplitude terms, the *k*-space Green’s function equals the exponential term in (7). For it, the wavenumber *z*-component, *k_z_*, can be computed from the so-called dispersion relation:

(8) $$k_{z}=\sqrt{(2{k)}^{2}-k_{x}^{2}-k_{y}^{2}}=\sqrt{{\left(\frac{2\omega }{c}\right)}^{2}-k_{x}^{2}-k_{y}^{2}},$$

which links frequency and wavenumber. The factor 2 again accounts for the roundtrip. In the geophysics literature, this is referred to as the “exploding reflector model” [25].

From (8), the inverse scattering problem, formulated in the spatial frequency domain, can be derived as

(9) $$O\left({k_{x}}_{\;},\;k_{y},\;\;z_{0}\right)=S_{\mathrm{scat}}\left({k_{x}}_{\;},\;k_{y},\;\omega \right)\;\text{·}\;\exp\;\left\{+j\;k_{z}\;z_{0}\right\}$$

It can be interpreted as a wavefield extrapolation in the wavenumber domain [25]. The exponential term is the reconstruction kernel in the wavenumber domain.

A direct implementation of (9) is the Phase Shift Migration algorithm [12]. It consists of the following steps (more details can be found in [25]):

1. 2D Fourier transform the receive signal over aperture dimensions (*x*, *y*);
2. Shift the spectrum obtained in step 1 iteratively to target planes via phase shift proportional to depth $z_{0}$ (Equation (9));
3. Focusing: 2D inverse Fourier transform over *k_x_* and *k_y_* and sum over frequency.

As can be seen from (9), the reconstruction kernel now solely depends on *z* and ω, which is why this kind of reconstruction is computationally more efficient. A depth-by-depth reconstruction for each depth plane is now possible instead of a voxel-by-voxel reconstruction as in the Backprojection algorithm. 

More efficient still is the Range Migration algorithm. Its main idea is to perform focusing by means of a 3D inverse Fourier transform instead of a 2D inverse Fourier transform plus summation over frequency. For this, a rescaling of the wavefield data to depend on *k_z_* instead of ω is necessary. This is performed by an interpolation using the dispersion relation. The interpolation is termed “Stolt interpolation” after its inventor. Because of the 3D inverse Fourier transform, the whole reconstruction volume can be reconstructed in one step. For details on the algorithm, see [13,25].

When there is a heterogeneous background, an adjustment of the Phase Shift Migration algorithm to can be accomplished by adapting the reconstruction kernel as

(10) $$H\;({k_{x}}_{\;},\;k_{y},\;\;z_{0},\;\omega )=\exp\;\left\{+j\;k_{\mathrm{zi}}\;z_{0}\right\},$$

where the index *I* in *k_zi_* denotes the *i*-th material in the multilayer geometry and

(11) $$k_{\mathrm{zi}}=\sqrt{{\left(\frac{2\omega }{c_{i}}\right)}^{2}-k_{x}^{2}–-k_{y}^{2}}$$

Therefore, simply by adjusting *k_zi_*, the same line-by-line reconstruction can be performed as before.

For the Range Migration, a reconstruction per material can be performed (see, e.g., [25]). Therefore, both wavenumber domain algorithms perform faster than the Backprojection algorithm. However, they cannot deal with curved boundaries.

### 2.2. Proposed Approach

As mentioned, the idea of our concept is to form an image from a superposition of single sub-images where as much of the image volume as possible is reconstructed in the wavenumber domain and as little as possible is reconstructed in the spatial domain. For this, first, a segmentation has to be performed to determine which image parts can be reconstructed in the wavenumber domain and which have to be reconstructed in the spatial domain.

To determine which parts can be reconstructed in wavenumber domain, we regard Equation (10). Inherent in this reconstruction kernel, which is proportional only to *z*, is the assumption that the material is varying only along depth *z* but not along the lateral dimensions *x* and *y*. Therefore, the wavenumber domain reconstruction requires a planar material boundary. It cannot deal with irregular ones [21].

Accordingly, we have to find those image points whose optical paths will intersect with the planar boundary parts for all antenna positions. These image parts can be reconstructed in the wavenumber domain correctly because the underlying assumption of a planar material boundary will hold. Only the remaining part, which is influenced by the non-planar boundary part, must be reconstructed in the spatial domain. This segmentation is the core of our proposed hybrid concept.

**Figure 3:**
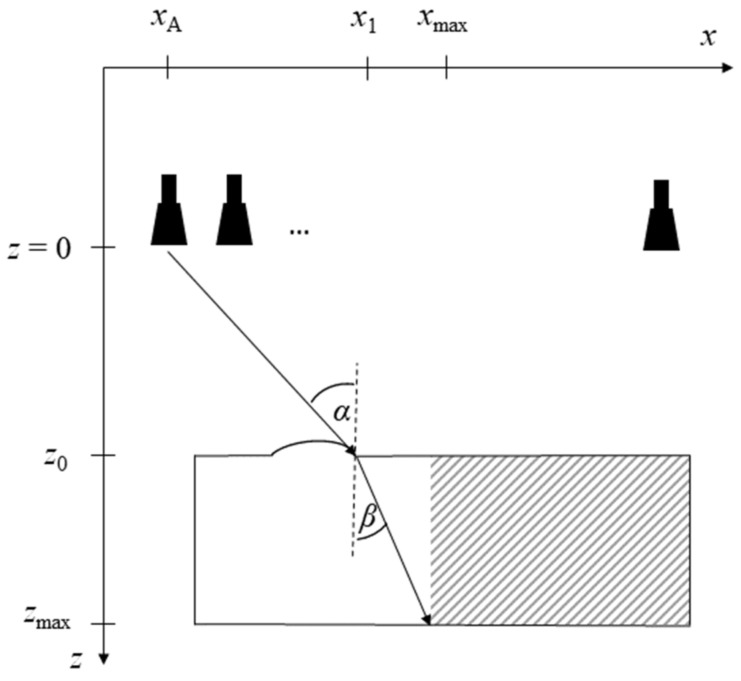
Example geometry sketch for determining the maximum refracted optical path. The incident and refracted angles are denoted *α* and *β*, respectively. The lateral dimension is *x*; the depth dimension is *z*. The hatched area represents the area to be reconstructed in *k*-space.

In order to find the image segment that can be reconstructed in the wavenumber domain, simple geometry can be used. This is illustrated in Figure 3, where a scenario with a partly planar material boundary is illustrated. Here, the boundary consists of a non-planar section between two planar ones. In the figure, the imaging geometry is sketched along with three antennas, representing the first, second, and last position of the synthetic aperture. The lateral dimension is denoted *x*; the depth dimension is denoted *z.*

At that boundary point that separates the planar and non-planar boundary parts (*x*_1_, *z*_0_), the maximum refracted optical path is computed. It obviously corresponds to the outermost antenna position, as illustrated in Figure 3.

For computation of the refracted path, the refractive index as well as the incident angle are used: the angle of incidence, *α*, is provided by the range *z*_0_ from the aperture plane and the distance between the outermost antenna and the intersection point (*x*_1_, *z*_0_) at the material boundary:

(12) $$\tan\;(\alpha )=\frac{x_{1}-x_{A}}{z_{0}}.$$

Using Snell’s law, (6), we can find the angle of refraction, *β*. The coordinate denoted *x*_max_ in Figure 3 is then determined using geometric relations:

*x*_max_ − *x*_1_ = tan (*β*) · (*z*_max_ − *z*_0_), (13)

The point (*x*_max_, *z*_max_) separates the image domain into the part that can be reconstructed in *k*-space and that to be reconstructed in the spatial domain. All points right of *x*_max_ (i.e., *x* ≥ *x*_max_; hatched area in Figure 3) can be computed in *k*-space. Since the antenna position with the maximally possible refraction will have an optical path through the planar boundary part, all other antennas—whose incident angles will be smaller—will also have an optical path through the planar boundary part. Thus, in total, the hatched region in Figure 3 can be reconstructed correctly in the wavenumber domain because the underlying assumption of a planar material boundary is satisfied. Only the area in white needs to be computed in the spatial domain.

Note that it would be possible to reconstruct all points right of the refracted line in the wavenumber domain. Merely for the sake of implementation simplicity, the region is chosen to be rectangular.

The same procedure needs to be performed for the other side of the curved boundary part. Then, the right-most antenna position must be evaluated, but the calculation is analogous to Equations (12) and (13). 

Note that Snell’s law as in (6) is valid only for real-valued refractive indices, i.e., lossless materials. Therefore, we restrict ourselves to lossless materials for our concept.

With the segmentation of the reconstruction domain, the overall image can then be reconstructed from superposing sub-images. To form the overall image, first, the whole volume is reconstructed in *k*-space. Then, the necessary part is reconstructed in the spatial domain and the first sub-image is overwritten in these regions.

When stitching the sub-images together, care must be taken regarding image amplitudes: the sub-images can possibly display different amplitude scales because they are reconstructed from two different algorithms. A possible approach to overcome this problem is to normalize both images to the amplitude of the boundary reflection since it will most likely be present in both sub-images.

In sum, the novel reconstruction concept consists of the following steps:

1. Divide reconstruction domains according to Section 2.2;
2. Sub-image 1: Reconstruct whole image in *k*-space;
3. Sub-image 2: Reconstruct necessary image part in spatial domain;
4. Adjust amplitudes by means of boundary reflection;
5. Overwrite necessary image part of sub-image 1 by sub-image 2.

For the reconstruction of sub-image 1, Phase Shift Migration or Range Migration can be used; for sub-image 2, Backprojection or others (Phase Shift Plus Interpolation, Split Step Migration, etc.). Detailed descriptions of the algorithms can be found in Section 2.1.

### 2.3. Discussion

It is possible that some image points within the hatched area in Figure 3 will be influenced by the planar boundary part as well as by the non-planar part. Nonetheless, the proposed concept still holds. We justify this by Fermat’s principle. This principle was originally formulated as the “principle of least time”. However, in its modern version, it states: when a wave travels from one point *P*_1_ to another point *P*_2_, the wave will take an optical path that is stationary w.r.t. the variation in that path. This can be expressed in the sense of the calculus of variation as [26]

(14) $$\delta l_{\mathrm{opt}}\;(x,\;y,\;z)=\delta \int_{P_{1}}^{P_{2}}n\;(x,\;y,\;z)\;ds=0$$

where *δl*_opt_ is the variation in the optical path, which is the geometrical path increment d*s* multiplied by the space-variant refractive index *n* (*x*, *y*, *z*).

Thus, Fermat’s principle version implies that there may be several valid solutions for the optical path [27]. Therefore, a solution through the planar boundary part may be one out of possibly several solutions. However, since there is a valid path through the planar boundary part, the assumptions for the *k*-space reconstruction are satisfied and the corresponding image section can be reconstructed in *k*-space correctly.

### 2.4. Experimental Verification

**Figure 4:**
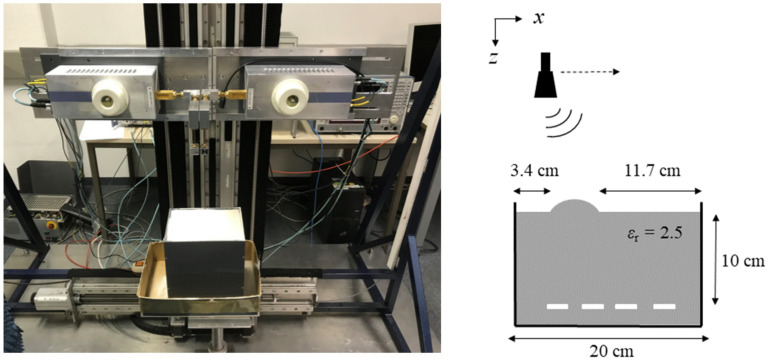
Test setup used for the experiments and sketch of setup with dimensions. A quasi-monostatic SAR illuminates a box filled with sand and four stripes of aluminum foil buried in it (displayed in white in the sketch).

Experiments with a quasi-monostatic, equidistantly sampled SAR were conducted to verify the introduced concept. The SAR system was implemented as a single-input–single-output (SISO) radar. A photograph of the employed laboratory setup is shown in Figure 4 along with a sketch showing the setup’s dimensions.

The experimental setup consists of a down-looking radar that is moved step-wise along a line on a traversing unit. The radar itself is a vector network analyzer (type Rohde & Schwarz ZVA 24) combined with frequency extenders. Linearly polarized horn antennas are used as transceivers. They emit and receive a stepped frequency continuous wave signal in the frequency range of 75 GHz to 110 GHz. The antennas were mounted to the frequency extenders on the traversing setup by means of 90-degree bends at a height of 20.3 cm above the sand’s surface.

A line scan with a total length of 18 cm was performed, sampled in steps of 1 mm. At each point, the frequency range was sampled in 401 steps. From the line scan, a 2D image can be reconstructed.

The SAR operates in a quasi-monostatic setup. The assumption of a quasi-monostatic setup is valid only for large distances w.r.t. the wavelength. In our case, for a maximum wavelength of 4 mm, the distance between the antenna aperture and the sand is more than 50 times the wavelength, which justifies the quasi-monostatic assumption. 

The employed test object is a plastic box (H × W × L: 20 cm × 15 cm × 20 cm) filled with sand. In the sand, four stripes of aluminum foil are buried, which are four targets to be imaged. Their width is approx. 1 cm.

To simulate an imaging geometry with a partly planar material boundary, the sand’s surface was flattened, and then a little hill was added. The resulting geometry, which is supposed to resemble the one in Figure 3, is shown in Figure 4.

**Figure 5:**
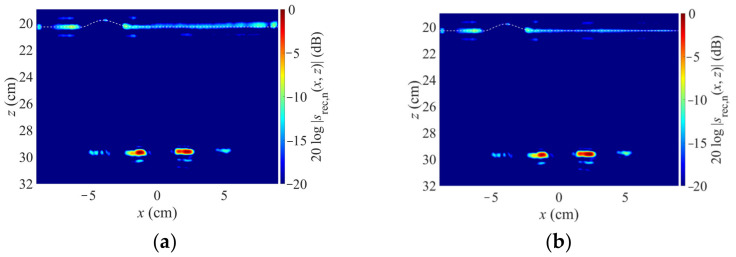
Image reconstruction by proposed hybrid concept (**a**) and state of the art (Backprojection with ray tracing, (**b**)).

For the reconstruction, the segmentation as described was performed. The result is shown in Figure 5: for the given geometry, only 47.22% of the image needs to be reconstructed in the spatial domain (white area in Figure 3). Accordingly, 52.78%—more than half of the volume—can be efficiently reconstructed in *k*-space (hatched area in Figure 3). Note that this percentage is geometry-dependent.

Figure 5a shows the image reconstructed from the experimental data by means of the proposed hybrid concept. Figure 5b shows the reference image reconstructed according to the state of the art, i.e., with the spatial domain Backprojection algorithm combined with ray tracing as described in Section 2.1. Both images are displayed in logarithmic scaling, normalized to their respective maximum values. 

For the reconstruction, the surface was extracted by means of a maximum search within a depth area in which we assume the surface to be, followed by a polynomial fit to smooth the surface and reduce the effect of outliers. The so-found curve is marked by the dashed white line in Figure 5. The sand’s relative permittivity is 2.5. Since the sand is dry, we made the assumption of a lossless material. 

It can be seen that the novel concept leads to some surface clutter, which might be due to a non-ideal amplitude adjustment of the two images, but there is no visible difference in the imaging of the targets. The proposed concept yields an image comparable to the one obtained with the state of the art. 

Therefore, it could be demonstrated that the proposed concept is feasible. Additionally, its computation time is much less than the reference image: when we assume that the fast Range Migration is used for the *k*-space reconstruction, a small two-dimensional geometry, as in the demonstration example, can be reconstructed in about real time. Since more than 50% of the image volume can be computed as fast, the reconstruction of the whole image takes approximately only half the time compared to the state of the art.

## 3. Extension to MIMO Radar

In security screening and non-destructive testing with high throughput requirements, MIMO radars are the state of the art. Therefore, the proposed concept is extended to MIMO radar imaging in this section. Note that SAR and MIMO are the same except that in SAR the antennas are moved, whereas in MIMO the frontend is stationary, composed of several antennas. The numerical aperture synthesis is the same principle in both cases.

### 3.1. Adaption of Proposed Concept

In the case of MIMO radar and 3D geometry, the segmentation of the reconstruction domain becomes more complex. In order to deal with it, we make an estimation here. For both dimensions *x* and *y* separately, we search for the maximally possible refracted optical path that occurs at the transition between planar and non-planar boundary parts. For determining this maximum refraction, again we have to consider the outermost antennas, which, this time, can be either transmitters or receivers. The outermost antennas again determine the maximally refracted optical path. Therefore, they are critical and determine the field that is influenced by the planar boundary part and therefore can be reconstructed in the wavenumber domain. 

Then, the procedure remains the same. As a *k*-space-based image reconstruction for an MIMO radar and a planar boundary, various algorithms can be employed (see, e.g., [15,16,28,29,30]). In this work, we used the algorithm described in [28]. For the space domain part, the Backprojection algorithm as described before was used.

### 3.2. Demonstration Setup

**Figure 6:**
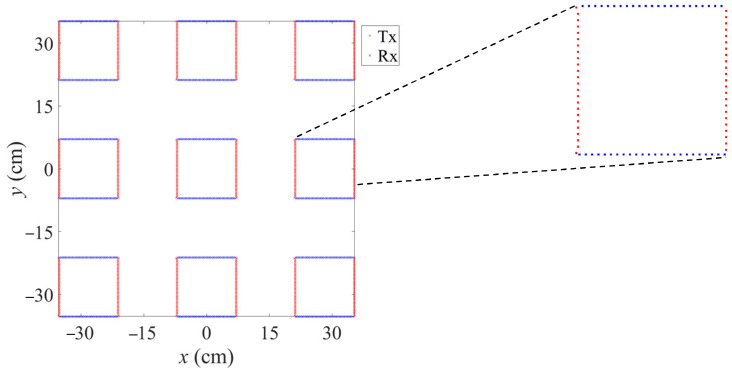
Employed sparse MIMO array. Transmitters are in red; receivers in blue.

As an MIMO imaging radar, we used a Rohde & Schwarz QAR scanner, and 846 transmit (Tx) and receive (Rx) antennas were used each. They are set up in a sparse array design as depicted in Figure 6. It consists of nine clusters, which are composed of two lines of 47 transmitters and 47 receivers each.

**Figure 7:**
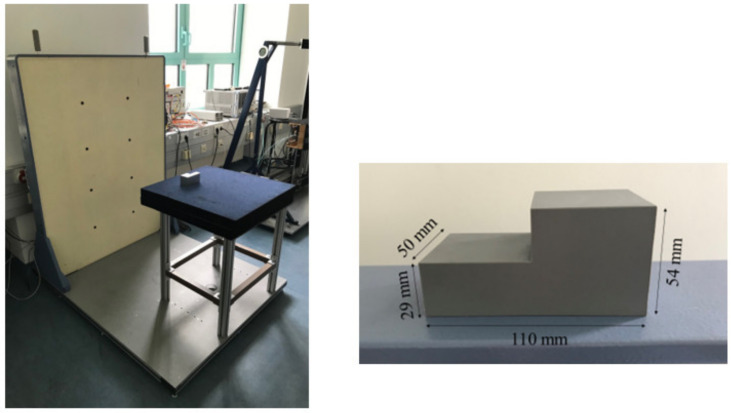
Photographs of measurement setup (**left**) and test object with dimensions (**right**).

The measurement setup and the employed test sample are shown in Figure 7. The sample under test is a polyvinylchloride (PVC, *ε*_r_ ≈ 2.7) object that has a surface composed of two heights. It was illuminated by the radar in such a way that the stepped profile represented the surface. During the measurement, the lower boundary was 65.3 cm away from the aperture plane and the upper boundary was 62.8 cm away.

### 3.3. Imaging Results

For using the proposed hybrid reconstruction concept, a segmentation of the reconstruction domains must be performed. The critical region here is the region around the middle of the stepped profile.

**Figure 8:**
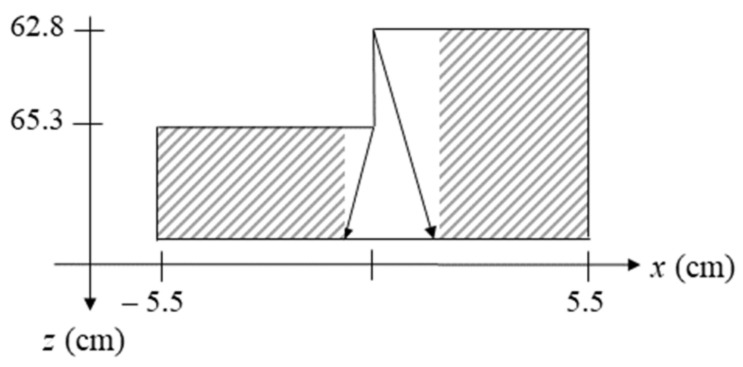
Segmentation according to the proposed concept. The arrows depict the maximally refracted optical paths at the boundary irregularity. They determine the image area to be reconstructed in *k*-space (hatched area).

For the two depths mentioned above (65.3 cm and 62.8 cm, respectively) and a maximum array length of 70.5 cm given by the scanner, the maximum possible angles of incidence are 29.1° and 28.2°, respectively. Using Snell’s law and the maximum image depth, the image segmentation can be obtained. It is shown in Figure 8. The arrows correspond to the maximum refracted optical paths; the hatched areas depict those areas that can be reconstructed in the wavenumber domain. Only the area depicted in white needs to be reconstructed in the spatial domain.

With the proposed concept, an image can thus be reconstructed from three sub-images:

- *k*-space reconstruction through planar material boundary part at *z* = 65.3 cm in the field *x* < −0.84 cm;
- *k*-space reconstruction through planar material boundary part at *z* = 62.8 cm in the field *x* > 1.56 cm;
- spatial domain reconstruction in the field −0.84 cm < *x* < 1.56 cm.

The field that needs to be reconstructed in the spatial domain equals 7 bins out of 235 along the *x*-axis. Note that the test object’s surface profile is constant along the *y*-axis, which is why no segmentation has to be performed along that domain.

**Figure 9:**
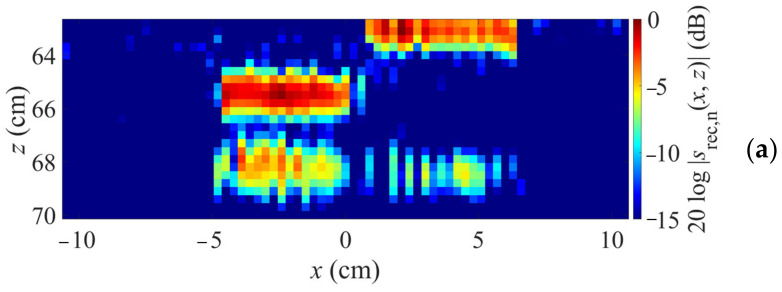

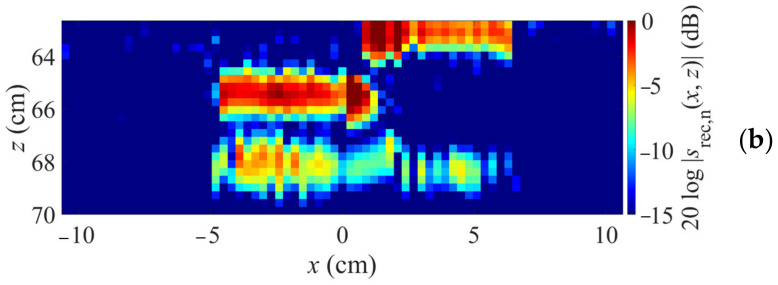
Image reconstruction by segmentation according to boundary parts (**a**) and proposed concept (**b**).

Reconstruction results are shown in Figure 9. Here, cross sections along *x* and *z* are shown. Figure 9a shows a simple segmentation according to the height profile, i.e., using two *k*-space reconstructions with planar material boundaries at 65.3 cm and 62.8 cm, respectively, for the regions below these boundary parts. It can be seen that, although the test object is recognizable, there is a gap in the lower boundary (at z ≈ 68 cm). In contrast, Figure 9b displays the image obtained when using the proposed concept. This time, the lower boundary is fully depicted, which verifies the approach also for MIMO imaging. 

### 3.4. Computational Complexity Analysis

To underline the efficiency of the proposed method, its computational complexity is compared to that of the standard Backprojection algorithm. Table 1 and Table 2 list the computational complexities of both algorithms’ single steps. See [28] for an explanation of the steps of the *k*-space-based MIMO reconstruction algorithm.

In the tables, *N_x_* and *N_y_* denote the number of image points along the lateral dimensions *x* and *y*; *N_z_* denotes the number of image points along the range dimension. The numbers of transmit and receive antennas are *N*_Tx_ and *N*_Rx_, respectively; *N_ω_* is the number of frequency steps. *N_x_*’ denotes the number of image points along the *x* direction that need to be reconstructed in the spatial domain.

It becomes clear that the proposed method has significantly lower complexity than the Backprojection algorithm. It amounts to a factor of approximately 32 for the demonstration example from Section 3.2 (*N*_Tx_ = *N*_Rx_ = 846 transmitters and receivers each, *N_ω_* = 64 frequency steps, *N_x_* = 235, *N_y_* = 235, *N_z_* = 35 image points in *x*, *y,* and *z* directions, respectively).

## 4. Conclusions

In this paper, a novel concept for subsurface radar imaging below a curved surface was introduced. The approach is based on the fact that many non-planar imaging geometries display a boundary that is partly planar. It was demonstrated that, in this case, efficient *k*-space-based reconstruction concepts for planar geometries can be applied for reconstructing parts of the subsurface image volume. That way, the computational efficiency of the image reconstruction can be significantly augmented while preserving an undisturbed image quality. The proposed concept was verified experimentally, both for monostatic SAR and MIMO radar.

At present, the approach is restricted to geometries that are large compared to the wavelength so that geometrical optics can be applied. Furthermore, the approach is formulated for lossless materials. Future work will investigate overcoming these restrictions.

## Figures and Tables

**Table 1:** Computational complexity of the proposed method.

Step	Computational Complexity
2D FFT	*N_x_* · *N_y_*· log_2_(*N_x_*·*N_y_*)
Backpropagation to boundary	*N_x_* · *N_y_* · *N_ω_*
Backpropagation inside material	*N_x_* · *N_y_* · *N_ω_*· (*N_z_* − 1)
2D IFFT	*N_x_* · *N_y_*· log_2_(*N_x_*·*N_y_*) · *N_z_*
Tx phase correction	*N*_Tx_ · *N_x_* · *N_y_* · *N_z_*· *N_ω_*
Summation ω	*N_ω_* · *N_x_* · *N_y_* · *N_z_*
Summation Tx	*N*_Tx_ · *N_x_* · *N_y_* · *N_z_*
Calculation of optical paths	*N*_Tx_ · *N_x_^’^*· *N_y_* · *N_z_ + N*_Rx_ · *N_x_^’^* · *N_y_* · *N_z_*
Spatial domain reconstruction	*N*_Tx_ · *N*_Rx_ · *N_x_^’^* · *N_y_* · *N_z_*· *N_ω_*
Total (*N*_Tx_ = *N*_Rx_*=* 846, *N_ω_* = 64, *N_x_ = N_y_* = 235, *N_z_* = 35, *N_x_*′ = 7)	2.74 · 10^12^

**Table 2:** Computational complexity of the Backprojection algorithm.

Step	Computational Complexity
Calculation of optical paths	*N*_Tx_ · *N_x_* · *N_y_* · *N_z_ + N*_Rx_ · *N_x_* · *N_y_* · *N_z_*
Spatial domain reconstruction	*N*_Tx_ · *N*_Rx_ · *N_x_* · *N_y_* · *N_z_* · *N_ω_*
Total (*N*_Tx_ = *N*_Rx_ *=* 846, *N_ω_* = 64, *N_x_ = N_y_* = 235, *N_z_* = 35, *N_x_*′ = 7)	8.85 · 10^13^

## Data Availability

The data presented in this study are available on request from the corresponding author.
